# What works – reaching universal HIV testing: lessons from HPTN 071 (PopART) trial in Zambia

**DOI:** 10.1097/QAD.0000000000001514

**Published:** 2017-06-28

**Authors:** Kwame Shanaube, Ab Schaap, Sian Floyd, Mwelwa Phiri, Sam Griffith, Joseph Chaila, Peter Bock, Richard Hayes, Sarah Fidler, Helen Ayles

**Affiliations:** a Zambart, Lusaka, Zambia; b London School of Hygiene and Tropical Medicine; c Imperial College, London, UK; d FHI 360, Durham, North Carolina, USA; e Department of Paediatrics and Child Health, Desmond Tutu TB Centre, Stellenbosch University, Cape Town, South Africa.

**Keywords:** community, HIV, home-based HIV counselling and testing, universal HIV testing and immediate treatment, Zambia

## Abstract

**Objective:**

To determine the uptake of home-based HIV counselling and testing (HCT) in four HPTN 071 (PopART) trial communities (implementing a ‘full’ combination HIV prevention package that includes universal HIV testing and treatment) in Zambia. We also explore factors associated with uptake of HCT in these communities.

**Design:**

HPTN 071 (PopART) is a three-arm community-randomized trial in 12 communities in Zambia and nine communities in South Africa evaluating the impact of a combination HIV prevention package, including universal HIV testing and treatment, on HIV incidence.

**Methods:**

Using a door-to-door approach that includes systematically revisiting households, individuals were offered participation in the intervention, and verbal consent was obtained. Data were analysed for the first 18 months of the intervention, December 2013 to June 2015 for individuals 18 years and older.

**Results:**

Among 121 130 enumerated household members, 101 102 (83.5%) accepted the intervention. HCT uptake was 72.2% (66 894/92 612), similar by sex but varied across communities. HCT uptake was associated with younger age, sex, community, being symptomatic for TB and sexually transmitted infections and longer time since previous HIV test. Knowledge of HIV status due to the intervention increased by 36% overall and by 66% among HIV positive participants; the highest impact was among 18–24 years old.

**Conclusion:**

Overall acceptance of HIV-testing through offering a door-to-door-based combination HIV prevention package was 72.2%. The intervention increased knowledge of HIV status from ∼50 to ∼90%. However, challenges still remain and a one-off intervention is unlikely to be successful but will require repeated visits and multiple strategies.

## Introduction

Significant progress has been made towards reducing the burden of the HIV epidemic globally and in sub-Saharan Africa (SSA). However, SSA remains the epicentre of the HIV epidemic accounting for almost 70% of new HIV infections globally [1]. In 2013, an estimated 24.7 million of the 35 million people living with HIV (PLWH) were in SSA, accounting for the highest number of PLWH globally [1]. In Zambia, HIV prevalence in the population aged 15–49 years is estimated to be 13.3% [2].

Voluntary HIV counselling and testing (HCT) has been shown to result in knowledge of one's HIV status and reduction in sexual risk behaviour in certain populations [3,4]. Knowing one's HIV status is the first step in accessing prevention and treatment services. HIV-positive individuals who do not know their status might unknowingly contribute towards onward HIV transmission and have a higher personal risk of developing opportunistic infections which could lead to early death [5].

Uptake of HIV testing has shown remarkable increases in the past 6 years (2009–2015) in Zambia. In the 2013–2014 Demographic Health Survey (DHS), the proportions of participants aged 15–49 years who have ever been tested for HIV was 80% in women compared with 64% in men [2]. However, the proportions of those tested in the previous 12 months remained quite low (women – 46%, men – 37%) [2]. Similarly, many countries in SSA show low levels of HCT uptake. According to a report using nationally representative data on HIV-testing, uptake from 47 surveys in 29 SSA countries that conducted DHS between 2003 and 2011, the percentage of participants aged 15–49 years who were ever tested for HIV and received results of the most recent test was 28.8% for women and 17.2% for men [6].

Modelling reports suggest that a ‘universal test-and-treat’ (UTT) [7] approach can provide significant HIV prevention benefits but only when very high levels of uptake of universal HIV testing and immediate treatment are achieved and sustained [8,9]. However, challenges of attaining high levels of UTT under routine settings have been widely acknowledged. Four large-scale cluster randomized trials in SSA to measure the feasibility and efficacy of a UTT approach in ‘real life’ [10–13] have provided the much needed evidence. One of these, the HPTN 071 trial, also known as PopART (Population Effects of Antiretroviral Therapy to reduce HIV Transmission) trial is a three-arm community randomized trial in 12 communities in Zambia and nine communities in South Africa evaluating the impact of a combination HIV prevention package, including UTT, on community-level HIV incidence over a 5-year period [12]. This trial is currently ongoing.

We report on the uptake of HCT in four HPTN 071 (PopART) trial communities (implementing a ‘full’ combination HIV prevention package that includes UTT through a door-to-door approach) in Zambia after the first 18 months (first round) of the intervention. We also explore factors associated with uptake of HCT in these communities.

## Methods

### Trial design

Full details of the HPTN 071 (PopART) design have been described elsewhere [12]. In brief, the 21 study communities were formed into seven matched triplets (four triplets in Zambia and three triplets in South Africa) and randomly allocated to three study arms. Arm A is receiving the full PopART intervention including immediate antiretroviral therapy (ART) irrespective of CD4^+^ cell count. The adult (≥18 years old) population of the four Arm A communities in Zambia and the three Arm A communities in South Africa is estimated to be approximately 104 000 and 55 000, respectively. The study is being carried out in areas known to have high HIV prevalence (approximately 15–20%) and is continuing to experience severe generalized HIV epidemics [12].

We report on the adult population aged 18 years and above from the four Arm A communities in Zambia only.

### The PopART intervention

The PopART combination HIV prevention package is delivered by a cadre of community workers called Community HIV Care Providers (CHiPs). All household members living in the intervention areas are offered the PopART intervention. The intervention includes ‘annual’ rounds of home-based HCT (HB-HCT) with linkage to prevention and care. Individuals found to be HIV-positive are referred to the local government clinics for linkage to HIV care and ART irrespective of CD4^+^ cell count with ongoing support for adherence and retention in care. Individuals receive information on HIV prevention and are offered condoms and screened for symptoms of tuberculosis (TB) and sexually transmitted infections (STIs). Symptomatic individuals for TB and STI are referred to the clinic for further management. Uncircumcised HIV-negative men are referred for voluntary medical circumcision (VMMC) at the clinic.

CHiPs are lay counsellors who work in pairs; each pair is allocated a zone of the community. A total of 103 CHiPs pairs work in the four intervention communities. The CHiP pair enumerate all household members and systematically visit all households in their zones to offer testing and retesting using HIV rapid tests. The teams return to households in their zones throughout the year as necessary to follow-up on referrals and linkages to care and offer testing to absent household members or those who previously declined testing. CHiPs record household activities on an electronic data capture (EDC) device.

### Consent to participate and to start ART regardless of CD4^+^ cell count

All household members are asked for verbal informed consent to take part in the intervention and permission to collect data on the EDC device. Consent to participate does not necessarily include consent for an HIV test, although this is encouraged. Information is given about the risks and benefits of UTT and participants give written informed consent to start ART outside national guidelines at the clinic.

### Home-based HIV counselling and testing

HCT is offered to all household members. Written consent for HCT is obtained from adults. Household members who agree to HCT have the option to receive it as couples, a household group, or individually. HIV testing is carried out using Alere Determine HIV-1/2 test (Alere International Limited, Matsudo-shi, Chiba, Japan) as a screening test and the Unigold HIV test (Trinity Biotech Manufacturing Ltd, Bray, Co. Wicklow, Ireland) as the confirmative test for individuals who have a reactive result on the screening test.

### Data collection

The household activities of CHiPs are electronically logged using a password-protected EDC device. Data collected include socio-demographic and clinical data which are synchronized daily to a central server. The EDC programme encrypts the names, addresses, and global positioning system-coordinates of the visited households to minimize the risk of identification of participants by individuals other than CHiPs.

### Statistical analysis

Data were collected from December 2013 to June 2015 and analysed using Stata V.13. Coverage of the intervention and possible biases in the inclusion of participants was explored by studying households consenting, age and sex distribution of those enumerated as a household member, age and sex distribution of those consenting to the intervention, and the proportion refusing or not contacted by CHiPs.

Analysis was restricted to participants aged 18 years and older, eligible for HIV-testing that is, all adults who did not self-report to be HIV-positive. Explanatory variables were limited to those variables collected by CHiPs and included community, age, sex, date previously tested for HIV, number of adults in the household, TB symptoms, STI symptoms, whether circumcised (men) or pregnant (women).

Univariable and multivariable random-effects logistic regression was used to estimate unadjusted and adjusted odds ratios (aOR), with all analysis controlled for community. Analysis was done separately for men and women, with zone as a random effect to account for clustering due to variation in CHiPs’ performance. The likelihood ratio test was used to compare the fit of different models and quantify evidence of associations between individual characteristics and HCT uptake. In multivariable models, age group and prior history of HIV-testing were considered for inclusion first, and confounding among community, age group, and previous history of HIV-testing was explored. Following this, other explanatory variables were considered and those that had weak evidence of association with the outcome (*P* < 0.10) were included, with robust standard errors to account for clustering by zone. Analysis was restricted to individuals with complete data.

Variables recorded by CHIPs on the EDC were selected primarily to support and monitor provision of the service by the CHiPs. *Knowledge of HIV status before the intervention* was defined as self-reported HIV-positive or tested HIV-negative elsewhere in the previous 12 months. *Knowledge of HIV status after intervention* was defined as self-reported HIV-positive and tested by CHiPs or tested HIV-negative elsewhere in the previous 12 months.

HIV-prevalence among participants was calculated using the formula: (self-reported HIV-positive participants) + (tested by CHiPs) + (estimated HIV-positive among those accepting the intervention but decline HIV-testing)/(all participants).

The estimated HIV-positive among those accepting the intervention but decline HIV-testing was calculated using predictions obtained from a multivariable logistic regression model whose outcome was HIV status, and including in the analysis all adults who did not self-report they were HIV-positive and accepted the offer of testing from CHiPs.

### Ethical approval

Ethics approval was obtained from the ethics committees of the University of Zambia (UNZA BREC) and the London School of Hygiene and Tropical Medicine. Permission to conduct the study was received from the Ministry of Health.

## Results

### Participation

**Fig. 1:**
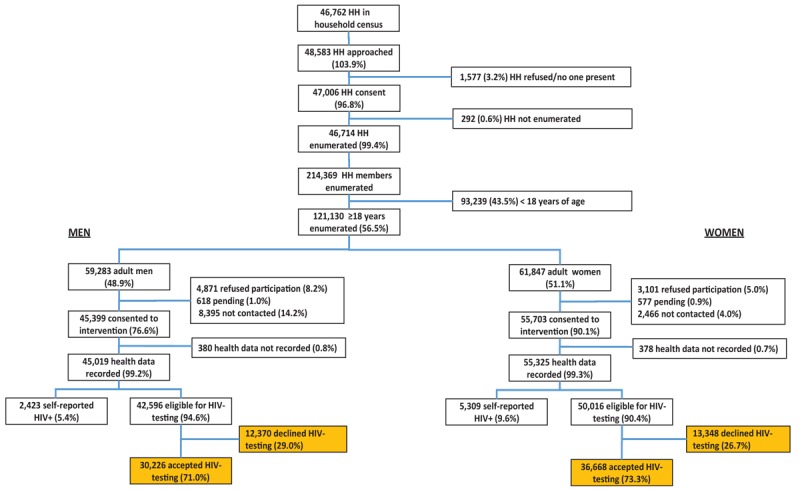
Flow chart showing PopART intervention participation and HIV-testing eligibility.

From December 2013 to June 2015, 48 583 households were visited. More households were visited than had been counted during household census in 2013 (46 762) due to new houses being built and additional households moving in (Fig. 1). Most households consented to the intervention (47 006/48 583; 96.8%). Enumeration of individual household members was completed for 99.4% (46 714/47 006) of the consenting households. Average household size was 4.6 (214 369 members in 46 714 households). Women were less likely to refuse to the intervention than men [5.0% versus 8.2%, aOR 0.55, 95% confidence interval (CI): 0.53–0.58]. CHiPs were not able to contact 14.2% of men and 4.0% women (Fig. 1).

HH, Households.

A total of 101 102 (83.5%; 101 102/121 130) household members consented (accepted) the intervention of whom 44.9% (45 399/101 102) were men, and 55.1% (55 703/101 102) were women. For 100 344 participants out of 101 102 (99.3%), health data were electronically recorded after accepting the intervention. In total, 7732 (7.7%; 7732/100 344) participants self-reported to be HIV-positive; 2423 men and 5309 women, leaving 92 612 participants eligible for HIV-testing (46% men, 54% women). Overall, 72.2% (66 894/92 612) participants accepted HIV-testing by the CHiPs. HCT uptake was similar by sex (73.3% women; 71% men).

### Factors associated with uptake of HIV counselling and testing

ORs were generally similar in univariable and multivariable analyses, showing little confounding. In multivariable analysis, factors strongly associated with HCT uptake were community, younger age, longer time since previous HIV test, and being symptomatic for TB or STI (Table 1).

**Table 1:** Factors associated with uptake of home-based HIV counselling and testing by sex.

Men										Women								
				Univariable^*^			Multivariable						Univariable^*^			Multivariable		
Characteristic	*N*	Accept HIV test	%	OR	95% CI	*P*	OR	95% CI	*P*	*N*	Accept HIV test	%	OR	95% CI	*P*	OR	95% CI	*P*
Total	42 596	30 226	71.0							50 016	36 668	73.3						
Community						<0.001			<0.001						<0.001			<0.001
1	5464	2833	51.8	0.32	0.19–0.54		0.26	0.16–0.44		6387	3994	62.5	0.47	0.29–0.76		0.39	0.25–0.63	
2	9319	7767	83.3	1.85	1.25–2.78		1.54	1.03–2.27		11 937	9907	83.0	1.75	1.22–2.56		1.69	1.19–2.44	
3	21 420	15 783	73.7	1.00			1.00			23 566	17 690	75.1	1.00			1.00		
4	6393	3843	60.1	0.48	0.32–0.72		0.55	0.36–0.82		8126	5077	62.5	0.47	0.32–0.69		0.53	0.36–0.76	
Age						<0.001			<0.001						<0.001			<0.001
18–19	4444	3672	82.6	1.00			1.00			6595	5474	83.0	1.00			1.00		
20–24	9135	7122	78.0	0.75	0.68–0.83		0.86	0.77–0.95		13 273	10 218	77.0	0.70	0.65–0.76		0.80	0.73–0.86	
25–29	7687	5461	71.0	0.51	0.47–0.57		0.67	0.60–0.74		9251	6599	71.3	0.53	0.49–0.58		0.61	0.56–0.67	
30–39	11 599	7701	66.4	0.41	0.37–0.45		0.54	0.48–0.60		11 010	7720	70.1	0.49	0.45–0.53		0.52	0.48–0.57	
40–49	5257	3434	65.3	0.38	0.35–0.43		0.46	0.41–0.51		4557	3174	69.7	0.47	0.43–0.51		0.42	0.38–0.47	
≥50	4474	2836	63.4	0.34	0.30–0.38		0.34	0.30–0.38		5330	3483	65.3	0.38	0.35–0.42		0.31	0.28–0.34	
Previous HIV test						<0.001			<0.001						<0.001			<0.001
Not tested	14 989	12 446	83.0	1.00			1.00			10 808	8877	82.1	1.00			1.00		
0–3 months	5970	2141	35.9	0.12	0.11–0.13		0.13	0.12–0.14		9405	4186	44.5	0.18	0.17–0.19		0.17	0.16–0.19	
4–6 months	4666	3029	64.9	0.43	0.40–0.47		0.46	0.42–0.50		7284	5294	72.7	0.63	0.58–0.68		0.61	0.56–0.65	
7–9 months	3094	2178	70.4	0.57	0.52–0.63		0.62	0.56–0.68		4702	3749	79.7	0.92	0.83–1.00		0.89	0.81–0.98	
10–12 months	2363	1709	72.3	0.64	0.57–0.71		0.68	0.61–0.77		3488	2845	81.6	1.03	0.93–1.14		1.01	0.91–1.12	
≥ 12 months	10 423	8020	76.9	0.79	0.74–0.84		0.88	0.81–0.94		13 074	10 849	83.0	1.14	1.05–1.22		1.18	1.10–1.27	
Unknown	765	588	76.9	0.56	0.46–0.68		0.65	0.53–0.80		1004	779	77.6	0.69	0.59–0.83		0.79	0.66–0.94	
Missing	326	115	35.3							251	89	35.5						
Household size						<0.001			<0.001						<0.001			<0.001
1 adult	2029	1583	78.0	1.00			1.00			2625	1893	72.1	1.00			1.00		
2 adults	15 727	10 241	65.1	0.55	0.49–0.61		0.61	0.54–0.69		20 100	14 225	70.8	0.96	0.87–1.05		0.89	0.80–0.99	
3 adults	8659	6198	71.6	0.79	0.69–0.88		0.76	0.67–0.88		10 304	7637	74.1	1.18	1.06–1.32		1.04	0.93–1.16	
>3 adults	16 181	12 204	75.4	0.99	0.88–1.11		0.89	0.78–1.02		16 987	12 913	76.0	1.30	1.19–1.45		1.16	1.04–1.28	
TB						<0.001			<0.001						<0.001			<0.001
Not symptomatic	41 843	29 571	70.7	1.00			1.00			49 423	36 151	73.1	1.00			1.00		
On treatment	113	76	67.3	0.74	0.48–1.14		1.33	0.83–2.13		69	51	73.9	1.18	0.67–2.08		1.54	0.85–2.78	
Presumptive TB case	640	579	90.5	3.85	2.94–5.00		4.55	3.33–6.25		524	466	88.9	2.94	2.22–4.00		3.03	2.22–4.00	
Pregnant															<0.001			
No										45 836	33 782	73.7	1.00					
Yes										3929	2672	68.0	0.71	0.65–0.76				
Missing										251	214	85.3						
STI						<0.001			<0.001						<0.001			<0.001
Not symptomatic	41 826	29 553	70.7	1.00			1.00			49 075	35 843	73.0	1.00			1.00		
Symptomatic	686	608	88.6	3.57	2.86–4.55		3.33	2.50–4.35		839	756	90.1	3.85	3.03–5.00		3.45	2.70–4.55	
Missing	84	65	77.4							102	69	67.6						
Circumcision						<0.001			<0.001									
Not circumcised	22 940	17 030	74.2	1.00			1.00											
VMMC	13 526	9254	68.4	0.68	0.63	0.73	0.85	0.79–0.93										
Traditional	5190	3437	66.2	0.81	0.63	0.85	0.88	0.83–0.93										
Missing	940	505	53.7															

95% CI, 95% confidence interval; OR, odds ratio.

^*^Adjusted for community and clustering per CHiP zone.

Compared with community 3, participants in community 2 were more likely to accept HCT (aOR 1.54; 95% CI: 1.03–2.27 in men, 1.69, 95% CI 1.19–2.44 in women), whereas those in community 1 were more likely to decline, particularly men (aOR 0.26, 95% CI: 0.16–0.44 in men, 0.39, 95% CI 0.25–0.63 in women).

Participants aged 18–19 years had the highest HCT uptake with a trend towards increasing refusal from ages 20 to 50. Participants aged 50 years and older were more likely to decline HCT (aOR 0.34, 95% CI 0.30–0.34 in men; 0.31, 95% CI 0.28–0.34 in women).

Participants recently tested for HIV had the lowest HCT uptake compared with those who never tested (aOR: 0.13, 95% CI 0.12–0.14 in men; 0.17, 95% CI 0.16–0.19 in women). Men living in households with one other adult member had lower HCT uptake (aOR 0.61, 95% CI 0.54–0.69) compared with a single adult man, whereas for women, difference in HCT uptake according to household size was small.

Being symptomatic for TB was the most noteworthy factor associated with higher HCT uptake, although a low percentage of the population reported TB symptoms. Compared with asymptomatic participants, those symptomatic for TB were more likely to accept HCT (aOR: 4.55, 95% CI 3.33–6.25 in men, 3.03, 95% CI 2.22–4.00 in women). A similar trend was seen for participants reporting STI symptoms (aOR: 3.33, 95% CI 2.50–4.35 in men; 3.45, 95% CI 2.70–4.55 in women). Evidence of an association between VMMC and HCT uptake was less convincing. Being pregnant was associated with lower HCT uptake in univariable analysis (OR 0.71, 95% CI 0.65–0.76), but there was no evidence of an association in multivariable analysis.

### Effect modification

Although there was evidence of effect modification between community, age, and previous HIV testing history, it was the strength of association that varied rather than the direction of association. For this reason, overall findings only are presented. As including interaction terms did *not* change the overall message, stratified ORs are not shown.

### Proportion of HIV-positive participants among those who consented

#### Self-reported HIV-positive participants

Overall, 5.4% (2423/45 019) of men and 9.6% (5309/55 325) of women self-reported to be HIV-positive, varying by community and age (Tables 2 and 3). The proportion of participants reporting to be HIV-positive increased with age for those aged 18–49. Most self-reported HIV-positive participants were diagnosed more than 12 months previously (13.9% men and 22.1% women). Expectedly, the highest proportion of self-reported HIV-positive status was seen in participants on TB-treatment (49.6% men and 57.1% women).

**Table 2:** Self-reported HIV-positive and newly HIV-positive identified by Community HIV care Providers in men.

Characteristic	Total consented to intervention	Self-reported HIV+	Tested HIV+	% Tested HIV+	Known HIV+ after intervention	Known HIV+ among consented after intervention (%)	Proportion HIV+ diagnosed by the CHiPs (%)	HIV-prevalence among consenters (%)
Total	45 019	2423	1715	5.7	4138	9.2	41.4	10.3
Community								
** **1	5640	176	74	2.6	250	4.4	29.6	5.6
** **2	9871	552	556	7.2	1108	11.2	50.2	12.2
** **3	22 635	1215	865	5.5	2080	9.2	41.6	10.2
** **4	6873	480	220	5.7	700	10.2	31.4	11.9
Age								
** **18–19	4461	17	14	0.4	31	0.7	45.2	0.7
** **20–24	9182	47	130	1.8	177	1.9	73.4	2.2
** **25–29	7848	161	330	6.0	491	6.3	67.2	7.3
** **30–39	12 540	941	764	9.9	1705	13.6	44.8	15.5
** **40–49	6109	852	335	9.8	1187	19.4	28.2	21.3
** **≥50	4879	405	142	5.0	547	11.2	26.0	12.3
Previous HIV test								
** **Not tested	14 989	0	818	6.6	818	5.5	100.0	6.5
** **0–3 months	6167	197	64	3.0	261	4.2	24.5	5.9
** **4–6 months	4844	178	117	3.9	295	6.1	39.7	7.2
** **7–9 months	3232	138	76	3.5	214	6.6	35.5	7.5
** **10–12 months	2498	135	86	5.0	221	8.8	38.9	10.0
** **≥12 months	12 104	1681	509	6.3	2190	18.1	23.2	19.1
** **Unknown	859	94	36	6.1	130	15.1	27.7	16.3
** **Missing	326	0	9	7.8	9	2.8	100.0	
Household size								
** **1 adult	2165	136	136	8.6	272	12.6	50.0	13.9
** **2 adults	16 980	1253	760	7.4	2013	11.9	37.8	13.5
** **3 adults	9117	458	308	5.0	766	8.4	40.2	9.4
** **>3 adults	16 757	576	511	4.2	1087	6.5	47.0	7.2
TB								
** **Not presumptive TB	44 049	2206	1526	5.2	3732	8.5	40.9	9.6
** **On treatment	224	111	21	27.6	132	58.9	15.9	62.1
** **Presumptive TB case	746	106	168	29.0	274	36.7	61.3	38.3
STI								
** **Not symptomatic	44 170	2344	1586	5.4	3930	8.9	40.4	10.0
** **Symptomatic	763	77	127	20.9	204	26.7	62.3	28.4
Missing	86	2	2	3.1	4	4.7	50.0	
Circumcision								
** **Not circumcised	24 772	1832	1382	8.1	3214	13.0	43.0	14.6
** **VMMC	13 829	303	172	1.9	475	3.4	36.2	3.8
** **Traditional	5411	221	132	3.8	353	6.5	37.4	7.7
** **Missing	1007	67	29	5.7	96	9.5	30.2	

CHiPs, Community HIV care Providers; HIV+, HIV-positive; STI, sexually transmitted infections; TB, tuberculosis.

**Table 3:** Self-reported HIV-positive and newly HIV-positive identified by Community HIV care Providers in women.

Characteristic	Total consented to intervention	Self-reported HIV+	Accept test	Tested HIV+	% Tested HIV+	Known HIV+ after intervention	Known HIV+ among consented after intervention (%)	Proportion HIV+ diagnosed by the CHiPs (%)	HIV-prevalence among consenters (%)
Total	55 325	5309	36 668	3393	9.3	8702	15.7	39.0	17.5
Community									
1	6769	382	3994	236	5.9	618	9.1	38.2	11.0
2	13 077	1140	9907	982	9.9	2122	16.2	46.3	17.5
3	26 074	2508	17 690	1617	9.1	4125	15.8	39.2	17.3
4	9405	1279	5077	558	11.0	1837	19.5	30.4	22.5
Age									
18–19	6675	80	5474	191	3.5	271	4.1	70.5	4.5
20–24	13 764	491	10 218	822	8.0	1313	9.5	62.6	10.8
25–29	10 149	898	6599	778	11.8	1676	16.5	46.4	18.8
30–39	13 307	2297	7720	1029	13.3	3326	25.0	30.9	27.4
40–49	5627	1070	3174	378	11.9	1448	25.7	26.1	27.9
≥50	5803	473	3483	195	5.6	668	11.5	29.2	12.9
Previous HIV test									
Not tested	10 810	0	8877	900	10.1	900	8.3	100.0	10.1
0–3 months	9846	441	4186	213	5.1	654	6.6	32.6	9.3
4–6 months	7647	363	5294	323	6.1	686	9.0	47.1	10.5
7–9 months	5030	328	3749	300	8.0	628	12.5	47.8	13.9
10–12 months	3811	323	2845	261	9.2	584	15.3	44.7	16.8
≥12 months	16 784	3710	10 849	1314	12.1	5024	29.9	26.2	31.4
Unknown	1145	143	779	65	8.3	208	18.2	31.3	19.6
Missing	252	1	89	17	19.1	18	7.1	94.4	
Household size									
1 adult	3235	610	1893	328	17.3	938	29.0	35.0	32.0
2 adults	22 545	2445	14 225	1452	10.2	3897	17.3	37.3	19.3
3 adults	11 337	1033	7637	651	8.5	1684	14.9	38.7	16.4
>3 adults	18 208	1221	12 913	962	7.4	2183	12.0	44.1	13.3
TB									
Not presumptive case	54 496	5073	36 151	3192	8.8	8265	15.2	38.6	16.9
On treatment	161	92	51	12	23.5	104	64.6	11.5	67.1
Presumptive case	668	144	466	189	40.6	333	49.9	56.8	52.7
Pregnant									
Not pregnant	50 788	4952	33 782	3129	9.3	8081	15.9	38.7	17.6
Pregnant	4260	331	2672	240	9.0	571	13.4	42.0	15.2
Missing	277	26	214	24	11.2	50	18.1	48.0	
STI									
Not symptomatic	54 220	5145	35 843	3189	8.9	8334	15.4	38.3	17.1
Symptomatic	1000	161	756	204	27.0	365	36.5	55.9	38.2
Missing	105	3	69	0	0.0	3	2.9	0.0	

CHiPs, Community HIV Providers; HIV+, HIV-positive; STI, sexually transmitted infections; TB, tuberculosis.

### Newly diagnosed HIV-positive participants

Among those who accepted the offer of HCT, 5.7% (1715/30 226) of men and 9.3% (3393/36 668) of women were newly diagnosed by CHiPs as being HIV-positive. The proportion of participants diagnosed as newly HIV-positive was lowest in the 18–19 age group (0.4% men; 3.5% women) (Tables 2 and 3). For both men and women, the highest proportion of newly diagnosed with HIV was found among those symptomatic for TB (29.0% in men and 40.6% in women) and STI (20.9% in men and 27.0% in women) as well as TB patients (27.6% in men and 23.5% in women).

Overall, the number of individuals known to have HIV increased by 66% as a result of the CHiPs intervention (7732 before versus 12 840 after). The relative benefit for the intervention varied between communities (varying from 29.6% in community 1 to 50.2% in community 2 in men and from 30.4 to 38.2% in women) and age groups [highest attribution was in the young age groups: 20–24 years (73.4%) in men and the age groups 18–19 (70.5%) in women].

### HIV-prevalence

Overall HIV-prevalence was higher in women than men (17.5% compared with 10.3%). Expectedly, the highest prevalence was found in those on TB treatment, with TB or STI symptoms, similar to the trend in the newly diagnosed HIV positives (Tables 2 and 3). Overall, prevalence estimates were higher in women for most risk-factor categories including age.

### Knowledge of HIV status before and after the PopART intervention

**Fig. 2:**
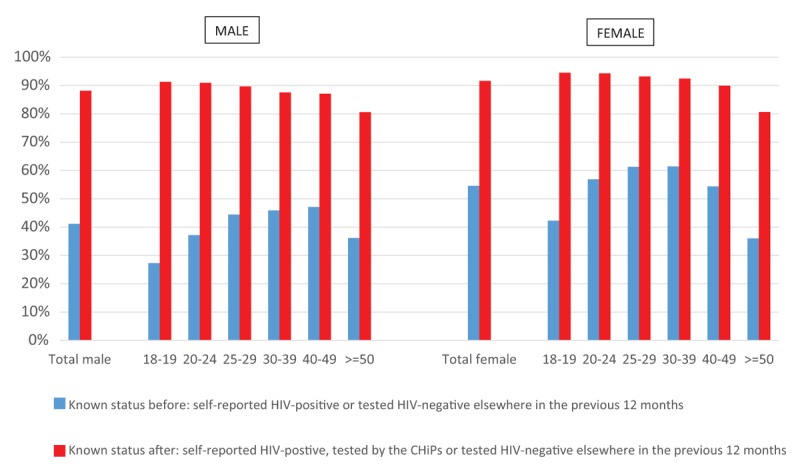
Knowledge of status before and after the intervention by sex and age.

Approximately, 41.1% of consenting men knew their HIV status before the intervention and this increased to 88.2% after the intervention. Similarly, approximately 54.6% of consenting women knew their HIV status before the intervention, and this increased to 91.6% after the intervention (Fig. 2).

The highest impact of the intervention in terms of knowing one's HIV-status was in the youngest age groups (18–19 years) in which knowledge of HIV status increased from 27.3 to 91.3% in men and from 42.2 to 94.5% in women.

### Additional value of repeated visits

Among those who accepted the offer of HCT, approximately 73.6% of women tested on the first visit to the household compared with only 52.7% in men. Among those who tested at any time during round one, 26.4% (9664/36 668) of women and 47.3% (14 282/30 2260) of men tested after the date their household was first visited.

Among those who accepted HCT at any time, the proportion of men who tested at a repeat visit, rather than the first visit to their household, was highest in those aged 40–49 years (52.7%) in men and those aged 18–19 years (33.7%) in women. The interquartile range across age groups was 44.7–52.7% for men and 22.0–33.7% for women (data not shown).

## Discussion

The current study provides important insight into the feasibility of delivering the first ‘90’ of the UNAIDS 90–90–90 targets under ‘real life’ conditions in SSA. To our knowledge, there are relatively few community-based population interventions exploring this to the magnitude of this study [14–16]. In contrast to other ongoing UTT trials, which were undertaken in smaller rural communities, we show that within a high HIV prevalence generalized epidemic in urban and periurban resource-limited settings, overall acceptance of HIV-testing through offering a door-to-door-based combination HIV prevention package was 72.2%. After one complete round of the PopART intervention, the percentage of participants who knew their HIV status increased from ∼50 to ∼90%, among those consenting, with the greatest impact being in the younger age groups.

The current study demonstrates that large-scale HB-HCT is well accepted, feasible, and effective in reaching the majority of resident population of a community consistent with findings from prior studies and other ongoing large-scale trials [13,16,17]. In addition, we demonstrate the additional value of revisits to increase coverage for HB-HCT to contact those who are absent or had previously declined testing. We show that for both men and women, repeat visits are important across the age range, and particularly necessary to reach men.

HCT uptake was associated with younger age, sex, and community, being symptomatic for TB and STI and longer time since previous HIV testing, consistent with findings from other studies [18–21]. Contextual heterogeneity has been shown to influence HCT uptake due to factors such as physical features, social organization, networks, and narratives [22]. Although the study communities were selected on the basis of common characteristics, they still have significant differences such as presence of other HIV testing options or stakeholders, scale of informal/formal populations, mobility patterns, quality of clinic services, and management of CHiPs’ teams. In communities 2 and 4, proportionally more adults took up the opportunity to be tested because of availability of several different HIV stakeholders levels and a high number of local HCT options [22]. In contrast, historically community 1 had fewer HIV efforts and has a stronger middle-income presence with more formal employment options which decreases the possibility of finding people at home [22].

HCT uptake was similar by sex, but it was much harder for CHiPs to contact men at home compared with women due to social-economic reasons such as beer-drinking and informal employment which takes them away from home [22]. Various initiatives such as community-based male health campaigns, weekend appointments, and HIV testing campaigns were tested to contact more men. These have been shown to increase HIV uptake [23,24].

Participants aged 50 years and older were more likely to decline HCT compared with the youngest age group. This is consistent with other studies that have found that such older age groups were associated with ‘never had an HIV test’ [6,21] despite being aware of an HIV testing site. In addition, we demonstrate that there is a relatively lower yield of new HIV-positive diagnoses amongst those 50 years and above and therefore they may not be a priority screening group. Uptake of HCT was high among women due to prior increased testing access at antenatal clinics before the CHiPs visit. Contrary, studies have shown that HCT uptake is lower in men regardless of the type of HCT offered [6,20,21].

Participants reporting symptoms of STI and/or TB had higher HCT uptake compared with asymptomatic ones probably due to perception of risk. According to the 2013–2014 DHS in Zambia, respondents with an STI or STI symptoms in the previous 12 months were more likely to be HIV-positive than those without (25 versus 14%) [2]. Similarly, in the Zimbabwe DHS men had higher odds of HCT uptake if reporting an STI in the previous 12 months (aOR 1.86; 95% CI: 1.26–2.74), whereas for women, visiting ANC [aOR 5.48; 95% CI (4.08–7.36)] was the most significant predictor of being tested [5].

The current study is being implemented under ‘real life’ conditions using a very large cadre of local community health workers. The survey design and sampling enables us to draw conclusion which should be broadly generalizable to other periurban/urban settings. However, the intervention is resource intensive, and it may be more feasible to deliver streamlined and targeted approaches.

Our analysis has distinguished the contributions of age, community, and previous testing and actually found relatively little confounding although these three factors are associated with each other and with HCT uptake. The factors explored in this article are not exhaustive as other important factors have been reported elsewhere [20,25]. We did not ask about reasons for declining HCT in the first round of the intervention but have subsequently done so in the second round. In a nested case–control study to examine factors associated with acceptance of HB-HCT delivered by CHiPs in the HPTN 071 trial, data from 642 participants showed no differences between cases and controls by demographic or behavioural characteristics (unpublished work).

We suggest that a UTT approach, delivered with the support of HB-HCT, should help in delivering the UNAIDS 90–90–90 targets designed to bring the global HIV epidemic under control.

### Conclusion

At a time when UTT is thought to be an effective strategy to eliminate HIV transmission, additional and innovative strategies will be required to reach ‘universal HIV testing’. By offering a HB-HCT programme, the uptake of testing can be increased to 72.2%, but challenges still remain in finding men and a one-off intervention is unlikely to be successful but will require repeated visits and multiple strategies.

## Acknowledgements

HPTN 071 is sponsored by the National Institute of Allergy and Infectious Diseases (NIAID) under Cooperative Agreements UM1-AI068619, UM1-AI068617, and UM1-AI068613, with funding from the US President's Emergency Plan for AIDS Relief (PEPFAR). Additional funding is provided by the International Initiative for Impact Evaluation (3ie) with support from the Bill & Melinda Gates Foundation, as well as by NIAID, the National Institute on Drug Abuse (NIDA) and the National Institute of Mental Health (NIMH), all part of the US National Institutes of Health (NIH).

Authors’ contributions: K.S. took the lead on writing the article. A.S. led on the statistical analysis and contributed to the writing of the article. All other authors commented on the article and approved the final version. The content is solely the responsibility of the authors and does not necessarily represent the official views of the NIAID, NIMH, NIDA, PEPFAR, 3ie, or the Bill & Melinda Gates Foundation. We are grateful to all members of the HPTN 071 (PopART) Study Team, and to the study participants and their communities, for their contributions to the research.

### Conflicts of interest

There are no conflicts of interest.
